# Non‐Linear Kinetics of The Lithium Metal Anode on Li_6_PS_5_Cl at High Current Density: Dendrite Growth and the Role of Lithium Microstructure on Creep

**DOI:** 10.1002/advs.202302521

**Published:** 2023-05-23

**Authors:** Dheeraj Kumar Singh, Till Fuchs, Christian Krempaszky, Boris Mogwitz, Jürgen Janek

**Affiliations:** ^1^ Institute of Physical Chemistry Justus‐Liebig‐University Giessen Heinrich‐Buff‐Ring 17 D‐35392 Giessen Germany; ^2^ Center for Materials Research (ZfM) Justus‐Liebig‐University Giessen Heinrich‐Buff‐Ring 16 D‐35392 Giessen Germany; ^3^ Institute of Materials Science and Mechanics of Materials Technical University of Munich Boltzmannstrasse 15 D‐85748 Garching Germany

**Keywords:** dendrite growth, lithium metal anodes, microstructure assisted creep, non‐linear kinetics, solid‐electrolyte interphase

## Abstract

Interfacial instability, viz., pore formation in the lithium metal anode (LMA) during discharge leading to high impedance, current focusing induced solid–electrolyte (SE) fracture during charging, and formation/behaviour of the solid–electrolyte interphase (SEI), at the anode, is one of the major hurdles in the development of solid‐state batteries (SSBs). Also, understanding cell polarization behaviour at high current density is critical to achieving the goal of fast‐charging battery and electric vehicle. Herein, via in situ electrochemical scanning electron microscopy (SEM) measurements, performed with freshly deposited lithium microelectrodes on transgranularly fractured fresh Li6PS5Cl (LPSCl), the LiǀLPSCl interface kinetics are investigated beyond the linear regime. Even at relatively small overvoltages of a few mV, the LiǀLPSCl interface shows non‐linear kinetics. The interface kinetics possibly involve multiple rate‐limiting processes, i.e., ion transport across the SEI and SE|SEI interfaces, as well as charge transfer across the LiǀSEI interface. The total polarization resistance *R*_P_ of the microelectrode interface is determined to be ≈ 0.8 Ω cm^2^. It is further shown that the nanocrystalline lithium microstructure can lead to a stable LiǀSE interface via Coble creep along with uniform stripping. Also, spatially resolved lithium deposition, i.e., at grain surface flaws, grain boundaries, and flaw‐free surfaces, indicates exceptionally high mechanical endurance of flaw‐free surfaces toward cathodic load (>150 mA cm^−2^). This highlights the prominent role of surface defects in dendrite growth.

## Graphical abstract

Lithium microelectrodes deposited on Li_6_PS_5_Cl are used to study a) the polarization behavior at high current density and the intrinsic interfacial kinetics, b) the influence of the lithium microstructure on interfacial stability. By means of spatially resolved lithium electrodeposition at Li_6_PS_5_Cl grain surface flaws, grain boundaries, and on flaw‐free surfaces, the origin of the mechanical failure is explored.

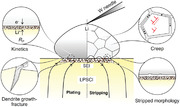

## 1 Introduction

The lithium metal anode (3860 mAh g^−1^, and *E*(Li^+^/Li) = −3.04 V vs standard hydrogen electrode), when coupled with inorganic solid electrolytes (SEs), can potentially enable solid‐state batteries (SSB) with high specific energy (≈393 Wh kg^−1^) and energy density (≈1143 Wh L^−1^) compared to conventional lithium‐ion batteries with liquid electrolytes.^[^ ^1^, ^2^, ^3^ ^]^ They also promise improved safety by eliminating thermal runaway issues associated with flammable liquid organic electrolytes, ensuring extended cycle life.^[^ ^4^, ^5^, ^6^ ^]^ However, practical applications, such as hybrid/electric vehicles, require that a high energy density is complemented by high‐rate capability.^[^ ^7^, ^8^ ^]^ Yet the reported rate capabilities of liquid electrolyte‐based batteries are still generally higher than those of SSBs.^[^ ^9^, ^10^ ^]^ Particularly, the lithium metal anode appears to be a critical limiting factor for a high power density of SSBs.^[^ ^11^ ^]^

The high‐rate capability of conventional LIBs is achieved through intercalation‐type electrodes that show only minor morphological and microstructural effects during charge and discharge, except under more severe conditions. The comparably low‐rate capability of the lithium metal anode is due to its inherent morphological instability during cycling—nucleation, growth, and the related massive volume effects remain key issues. Lithium deposition during charging can build up local interfacial stresses up to the GPa range.^[^ ^12^, ^13^ ^]^ It can easily exceed the fracture toughness (*K*_IC_) of ISEs, leading to crack initiation and subsequent filling with lithium.^[^ ^14^ ^]^ The crack propagation and resulting lithium dendrite growth clearly depend on the solid–electrolyte (SE) microstructure.^[^ ^15^, ^16^, ^17^, ^18^ ^]^ Therefore, understanding the micromechanics of dendrite growth is essential to address the low critical current density (CCD) observed in SSBs with the metal anode. This is particularly relevant for Li_6_PS_5_Cl (LPSCl), as the formation of interfacial contacts accompanies the introduction of flaws and microstructural restructuring due to the low *K*_IC_ of sulfur‐based SEs.^[^ ^17^, ^19^ ^]^

The formation of pores in the LMA during discharge additionally affects the power density.^[^ ^10^ ^]^ Therefore, maintaining a stable, low‐impedance anode interface is critical for the development of SSBs. To achieve this, usually, a high stack pressure is required.^[^ ^20^, ^21^ ^]^ As high pressure has a detrimental effect on cell performance when LPSCl is used as SE,^[^ ^22^, ^23^ ^]^ the resulting mechanical state of the lithium anode has an impact on the long‐term cyclability.^[^ ^12^, ^13^, ^24^ ^]^ It is desirable that this pressure threshold is as low as possible.

Beyond these electrochemo‐mechanical issues, the quantitative evaluation of the total polarization resistance (*R*_P_) at the Li|ISE interface is fundamental to the development of high‐rate SSBs.^[^ ^2^ ^]^ Polarization should be minor, preferably in the single digit Ω cm^2^ range, thus resulting in negligible area specific resistance (ASR). However, the presence of interface impurities (e.g., from cell fabrication or due to the passivation layer of stored lithium metal, and/or the formation of a solid–electrolyte interphase (SEI)), constriction effects (due to insufficient stack pressure or pore formation during discharge), hinder the determination of the intrinsic charge‐transfer resistance, *R*_CT_, the ion‐transport resistance across the SEI (*R*_SEI_) and the charge‐transfer resistance *R*_SE|SEI_ of the SE|SEI interface.^[^ ^25^, ^26^, ^27^, ^28^ ^]^ Correct interpretation of impedance data and assignment of microscopic transport/transfer steps in the low frequency range can get difficult due to current focusing (i.e., constriction), as recently shown by Eckhardt et al.^[^ ^29^ ^]^ To better understand the LMA quantitatively, it is important to deconvolute *R*_CT_, *R*_SEI_ and *R*_SE|SEI_ from the total polarization resistance *R*_P_ and address them accordingly. This is the especially important in the context of LPSCl as it forms a Li_2_S‐rich, self‐limiting, resistive SEI,^[^ ^30^ ^]^ with poor bulk ionic conductivity (*σ*_ion_(Li_2_S) ≈10^−10^ mS cm^−1^ at 25 °C),^[^ ^31^ ^]^ or an order higher conductivity for nanocrystalline Li_2_S depending on SEI microstructure.

In the present study, we employ freshly deposited lithium at a tungsten microneedle on the pristine surface of a transgranularly fractured LPSCl pellet, serving as a clean and well‐defined microelectrode—following a route first demonstrated by Krauskopf et al.^[^ ^32^ ^]^ This allowed us to study the following: i) polarization behavior of the LiǀLPSCl interface at high current density (40 mA cm^−2^), i.e., in the non‐linear regime. The low CCD values in SSBs occlude the study of polarization behavior at high current densities which are relevant for fast charging batteries in electric vehicles. ii) Microstructural influence of lithium on creep towards a stable, low‐impedance interface, and iii) identify the role of microstructure (i.e., grain surface, grain surface defects, or grain boundaries of LPSCl) in dendrite growth.

Under these model‐type conditions, we determine *R*_P_ ≈ 0.8 Ω cm^2^ by cyclic voltammetry, corresponding to a quite high exchange current density of *i*_0_ (Li|LPSCl) = 32 mA cm^−2^. We show that nanocrystalline lithium (<100 nm grain size) can reduce the required stack pressure to maintain a stable interface. Fast lithium diffusivity along low activation energy grain boundaries (*D*_GB_ ≈10^−7^ cm^2^ s^−1^), in a dense grain boundary microstructure enables a higher strain rate. In addition, microelectrode measurements on a defect‐free atomically flat grain surface can offer access to the theoretical strength (∼*E´*/15, where *E´* is Young's modulus of a given ceramic material) of the SE.^[^ ^33^ ^]^ Preferential lithium deposition (on defective and defect‐free grain surfaces) indicates an exceptionally high mechanical endurance of the defect‐free surfaces toward cathodic loading (>150 mA cm^−2^). This underlines the prominent role of surface defects and grain boundaries in dendrite growth.

## 2 Results and Discussion

LPSCl was synthesized via a solid‐state approach (see Supporting Information for details). The phase purity of the synthesized material was confirmed by powder X‐ray diffraction (PXRD) measurements (Figure S1, Supporting Information). Scanning electron microscopy (SEM) analysis reveals the grain size in the range of 20–40 µm (Figure S2, Supporting Information). The ionic conductivity of LPSCl pressed pellets was determined to be *σ*_ion_ = 1.65 mS cm^−1^ in a symmetric Au|LPSCl|Au cell using potentiostatic electrochemical impedance spectroscopy (PEIS; see Supporting Information for details). The activation energy of *σ*_ion_ was determined to be *E*_A_ = 0.40 eV by temperature‐dependent measurements in the range from −60 to +25 °C (Figure S3, Supporting Information).

**Figure 1:**
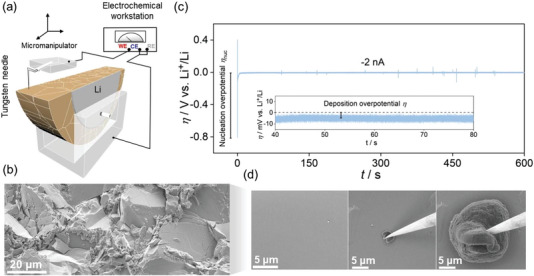
a) Experimental set‐up used for in‐situ SEM‐electrochemical measurements (schematic). b) SEM image showing surface topography of cross‐section of a cleaved LPSCl pellet with randomly oriented, transgranularly fractured grains. c) Galvanostatic profile at −2 nA for the first 10 min of deposition. d) Flat LPSCl grain surface before deposition (left). Temporal evolution of Li electrodeposition into prolate‐hemispheroidal deposits (middle and right).

The set‐up used for the in‐situ measurements in the SEM is schematically shown in **Figure** **1**a (see Supporting Information for details). A low magnification SEM image of the setup is shown in Figure S4 (Supporting Information). Figure S5a (Supporting Information) shows the FIB‐SEM image of the Li|LPSCl interface. In earlier work, we showed that an Li|LPSCl interface prepared in this way does not exhibit measurable constriction or charge‐transfer resistances.^[^ ^17^ ^]^ Figure S5b‐d (Supporting Information) shows the energy dispersive X‐ray spectroscopy (EDS) elemental mapping of S, P, and Cl at the interface. Figure 1b shows the surface topography of a cleaved LPSCl pellet with transgranularly fractured grains. To obtain a well‐defined lithium microelectrode, i.e., ideally hemispheroidal deposits, we applied the following current sequence: −2 nA for 10 min, −3 nA for 50 min, and −4 nA for 30 min (corresponding to ≈1250 µm^3^ lithium, i.e., ideally a semi‐sphere with *d* ≈ 10.6 µm). Figure 1c shows the galvanostatic deposition profile for the first 10 min showing the nucleation overpotential (*η*_nuc_ ≈ −800 mV) followed by a stable deposition overpotential of about *η* ≈ −4 mV. Figure 1d shows an SEM image of a flat LPSCl grain surface (left) along with the temporal evolution of the microelectrode at 30 s (middle) and 90 min (right).

### 2.1 Kinetics at LiǀLPSCl Interface

**Figure 2:**
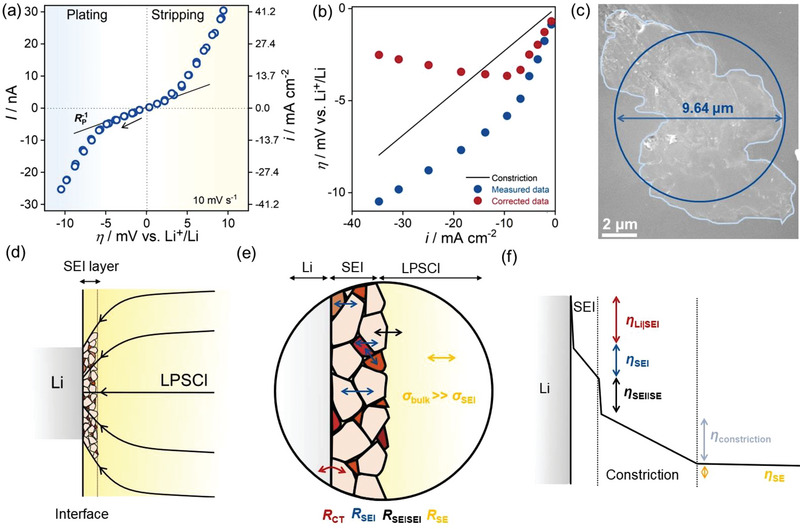
a) Cyclic voltammogram of a Li microelectrode at 10 mV s^−1^. The arrow indicates the initial scan direction. b) Cathodic overpotential corrected for constriction overpotential, i.e., I × Z_constriction_ = I × 1/(2*σ*d). c) The trace indicates the contacted area after microelectrode removal. The equivalent area with circular contact is also indicated. It corresponds well to *d* = 10.6 µm as estimated from the microelectrode deposition. d) Schematic depicting the SEI layer in the constricted electrode. Arrowed black lines schematically indicate the idealized current distribution in the SE‐SEI phase. In reality, the current distribution is expected to depend in a complex way on the microstructure of SEI. d) SEI as a multiphase composite with complex microstructure having different types of interfaces. Ion transport passes different interfaces, shown in blue. Additionally, ion transport across the SE|SEI interface and the charge transfer across Li|SEI. f) Plausible sources of polarization at the Li|LPSCl interface. The magnitude of the individual polarization can only be accurately estimated if the time constants of the processes are well separated.

Cyclic voltammograms (CVs) were recorded to evaluate the kinetic parameters of the Li|LPSCl interface. The CV at 10 mV s^−1^ (**Figure** **2**a) shows that the LiǀLPSCl interface exhibits non‐linear behavior. To understand the underlying kinetics, the interface overpotential at the working electrode must be deconvoluted from the total overpotential. Due to the asymmetric nature of the setup and the negligible interface resistance (constriction and/or charge‐transfer resistance) at the much larger counter/reference electrode,^[^ ^17^ ^]^ we assume that the potential drop occurs primarily at the microelectrode interface—a constricted electrode contact.^[^ ^26^, ^27^, ^28^, ^32^ ^]^ The impedance of a constricted electrode (*Z*_constriction_) of diameter *d* is determined under simplifying assumptions by the ionic conductivity *σ*_ion_ of the SE and is given by^[^ ^27^ ^]^

(1) $$Z_{\mathrm{constriction}}\approx 1/\left(2\times \sigma_{\mathrm{ion}}\times d\right)$$

Figure 2b shows the experimental data corrected for the constriction impedance, i.e., *η*_interface_ (interface overpotential) = *η*_measured_ (measured overpotential) − *I* × 1/(2×*σ*_ion_×*d*), where *I* is the current. We used the cathodic (plating) part of the CV for the analyses as the anodic data, particularly at high overpotentials (*η* > 5 mV) these seem to be slightly skewed due to contact loss at the interface. This is evident when comparing the extent of the linear regime in the cathodic and anodic scans. The linear regime extends until |*η*| ≈ 5 mV in the cathodic scan, whereas it is restricted to ≈2 mV for the anodic scan (stripping) (Figure 2a). The contact area of the working electrode was determined by following the trace of the microelectrode after its removal, as shown in Figure 2c. Then, the average diameter, *d* = 9.64 µm, was determined using a circular contact with an equivalent area (Figure 2c). Figure 2b shows that for the corrected data, the overpotential (|*η*|) first increases with increasing current density up to |*i*| = 9.4 mA cm^−2^ and then decreases with increasing current density. Clearly, this does not agree with our assumption of a simple constriction impedance, i.e., *Z*_constriction_ ≈ 1/(2×*σ*_ion_×*d*) does not hold. Since *d* is constant in our case (Figure 2c), the only parameter that could vary is *σ*_ion_, and we discuss this next.

### 2.2 Role of SEI

LPSCl forms a ≈250 nm thick Li_2_S‐rich resistive SEI with lithium (*σ*_ion_ (Li_2_S‐bulk) ≈10^−10^ mS cm^−1^ at 25 °C),^[^ ^31^ ^]^ see Figure 2d.^[^ ^30^ ^]^ The presence of other decomposition products, i.e., LiCl and Li_3_P, causes the formation of a multiphase composite with complex microstructure and multiple interfaces.^[^ ^27^ ^]^ In addition, ≈27% volume reduction occurs during the formation of SEI, according to the reaction Li_6_PS_5_Cl + 8Li → 5Li_2_S + LiCl + Li_3_P, indicating that the SEI may have a porous microstructure, unlike the dense SEI formed in Li‐ion batteries with liquid electrolytes, which are mainly composed of inorganic decomposition products (i.e., Li_2_CO_3_, LiF, etc.), with a thickness of ≈5–20 nm.^[^ ^34^, ^35^ ^]^

It is obvious that the ionic conductivity of the SEI must be taken into account when estimating the constriction impedance (Equation 1), i.e., *σ*_ion_ must include the contribution from the SEI. Therefore, the current distribution in the SE–SEI region is likely to depend on the microstructure (grain size, distribution of different phases, crystallinity of phases, porosity, etc.) and the thickness of the SEI.^[^ ^36^, ^37^ ^]^ This is shown schematically in Figure 2d–e. Figure 2d schematically shows the current distribution in the SE–SEI region. The current distribution will be highly inhomogeneous, and the subject deserves further theoretical and experimental investigation. Furthermore, if we assume that *σ*_ion_(SE–SEI) is an average value of *σ*_ion_(SEI) and *σ*_ion_(SE) that determines the constriction resistance, even then the overpotential *η*_constriction_ due to constriction is a linear function and cannot explain the observed non‐linearity. This will be discussed in the following section.

### 2.3 Non‐Linear Transport in Thin, Disordered SEI

Blume et al., and Hess, in their study of the interface between Li and solid‐polymer electrolyte (SPE), and alkali metals (Li, Na, and K) with various liquid electrolytes, respectively, pointed out that the non‐linear behavior of such interface, involving an SEI, cannot be described by simple Butler–Volmer (BV) kinetics.^[^ ^38^, ^39^ ^]^ Instead, they found more than one rate‐limiting process that determines the kinetics of the interface. This was confirmed by Farrington et al. in their lithium microelectrode kinetics study of liquid poly(ethylene glycol dimethyl ether) (PEGM) with LiAsF_6_.^[^ ^40^ ^]^ They showed that a SEI‐free interface can be described by BV‐type kinetics. Blume et al.  described their non‐linear kinetics with three rate‐limiting processes, viz. one ion transport step across the SEI and two charge‐transfer processes at the interfaces of the SEI. Both Blume et al. and Hess invoked a functionally similar hyperbolic sine law^[^ ^38^, ^39^ ^]^

(2) $$i\;=i_{0}\;\sinh\left(\beta \eta \right)$$

where, *i*_0_ and *β* are fitting parameters, along with an additional BV model to fit their experimental data. Equation 2 is similar to^[^ ^37^ ^]^

(3) $$i\;=\;4zFan_{+}\nu e^{-\left(W/RT\right)}\sinh\left(azF\eta /LRT\right)$$

where *z* is the charge number of the cation, *a* is the half jump distance between the hopping sites, *n*_+_ is the concentration of the cation lattice defect, *ν* is the vibration frequency of the cations at their sites, *W* being the activation energy of the hopping mechanism, *L* is the thickness of the SEI, and *η* is the overpotential due to electric field within SEI. Equation 3 describes the high‐field kinetics when the ion transport through a single‐ion conductor is rate‐determining.

SEI growth at the Li|LPSCl interface is self‐limiting, diffusion‐controlled, and thus a time‐dependent process that can be described by a Wagner‐type model.^[^ ^41^, ^42^, ^43^ ^]^ Wenzel et al.  showed that SEI growth into LPSCl follows a parabolic rate law, i.e., the thickness of SEI *d*_SEI_ ∝ √*t*, where *t* is the time.^[^ ^43^ ^]^ Under the present experimental conditions, i.e., deposition for 90 min with ever‐increasing electrode contact area (Figure 1d), followed by immediate measurements, the SEI layer is likely to be thin, possibly in the single‐digit or lower double‐digit nm range, with highly disordered microstructure. This implies that a strong electric field forms across the SEI at high current density, leading to field‐dependent non‐linear transport in this thin, disordered system.^[^ ^44^ ^]^ We assume that it is very likely that this is one of the factors contributing to the observed non‐linearity, and that the SEI cannot be treated as a linear resistive element. In addition, by studying the temperature dependencies of *β* and *i*_0_ (Equation 2), Blume et al.  argued that two additional processes, i.e., charge‐transfer across the Li|SEI and SE|SEI interfaces determine the kinetics, together with non‐linear ion transport across the SEI.^[^ ^38^ ^]^ Under these conditions, polarization at the Li|LPSCl interface would then involve three analogous contributions: *η*_Li|SEI_, charge‐transfer across the Li|SEI interface; *η*_SEI_, ion‐transport across the SEI; and *η*_SE|SEI_, charge transfer across the SE|SEI interface, leading to *R*_p_ = *R*_bulk_ + *R*_constriction_ + *R*_SEI_ + *R*_SEǀSEI_ + *R*_CT_ with *R*_constriction_ >> *R*_bulk_. This is shown in Figure 2f. Note that the actual magnitudes of the resistances can only be determined by impedance measurements if the time constants of the processes are well separated and should be additionally verified by temperature‐dependent measurements.

It will be highly interesting to study the influence of a modified SEI with different conductivity on the observed non‐linear kinetics in the future. SEI modification aiming for improved anode stability is increasingly being studied,^[^ ^45^, ^46^ ^]^ however, we are not aware of any systematic study yet. We expect that the observed non‐linear behavior will strongly depend on the type of SEI.

At low overpotentials, all (non‐linear) resistances converge to an Ohmic (linear) form, and the total polarization resistance *R*_p_ of the interface in this linear regime is determined to be ≈0.8 Ω cm^2^ (Figure 2a). The rather narrow linear range indicates a thin SEI and the onset of its non‐linear transport behavior. It should also be noted that the effect of the finite needle load on the microelectrode kinetics must be considered. While a modified form of BV kinetics has been proposed to account for the effect of stress on interfacial kinetics, it is only applicable if the applied stress is above 10 MPa.^[^ ^47^ ^]^

**Figure 3:**
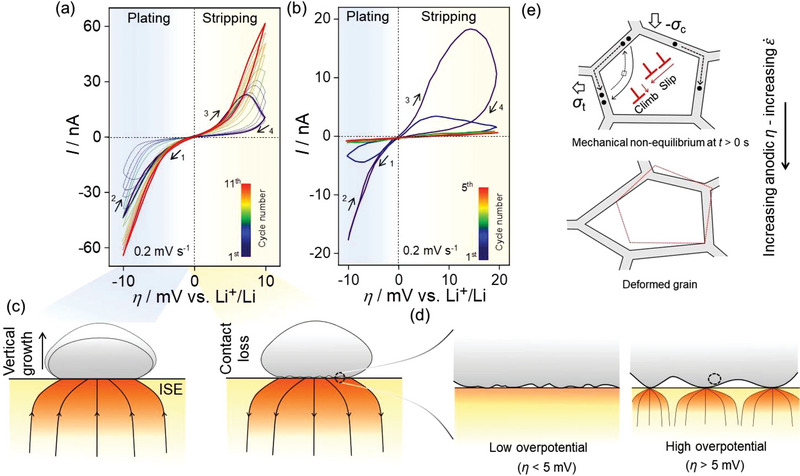
CVs of lithium microelectrodes. a) Symmetric, and b) asymmetric CV of the Li microelectrode at 0.2 mV s^−1^. The numbered arrows indicate the scan direction. c) Schematic of interfacial contact evolution during stripping and plating. Interfacial contact at high and low overpotential is expanded in (d). There are only a few contacts at high overpotentials compared to low overpotentials resulting in increased contact stresses. e) Creep deformation of a representative grain in the vicinity of the void (indicated by the dashed circle in (d)) subjected to time‐dependent or the anodic overpotential dependent inhomogeneous stress. Grain boundary diffusion indicated by solid circles or equivalently vacancy transport (indicated by square box) in the opposite direction drives creep, leading to interfacial contact healing. A net imbalance of stresses upon stripping drives deformation via dislocation climb creep (DCC) or glide assisted flow. The contour of grain boundary prior to deformation (depicted by the dashed red line) is overlayed on the deformed grain. *σ*_c_, compressive stress and *σ*_t_, tensile stress.

To evaluate the effect of the scan rate on the *I–η* response, CVs were recorded at a small scan rate of 0.2 mV s^−1^. The scan was initially set in the cathodic (plating) direction, as indicated by the arrow in **Figure** **3**a. Figure 3a shows the overlap between the cathodic currents in the forward and reverse scans for the first cycle. Upon switching to the anodic scan, symmetric *I‐η* behavior is observed up to *η* = 5 mV. Thereafter, a current drop is observed. When the anodic scan is reversed, the current continues to decrease steadily, resulting in current hysteresis. The interfacial contact situation leading to current hysteresis is schematically depicted in Figure 3c. Vertical Li growth leads to a morphologically stable interface during cathodic deposition. In contrast, anodic stripping causes a morphologically unstable interface via contact loss (Figure 3c). The resultant decrease in contact area leads to progressively decreasing current during the anodic scan (Figure 3a,c). We discuss the current hysteresis induced during anodic stripping and its subsequent closure (Figure 3a) by interfacial contact loss and contact healing, respectively, in the next section (Figure 3e).

The effect of anodic scan is carried over to the cathodic scan in the second cycle (Figure 3a), where it is seen that *I*_cat_(forward) < *I*_cat_(reverse). Furthermore, *I*_cat_(reverse) overlaps with the cathodic current in the first cycle. As the number of scans increases, we observed that *|I*_cat_(forward)| increasingly tends to *|I*_cat_(reverse)| or vice versa, i.e., *|I*_an_(reverse)| → *|I*_an_(forward)| until the 11^th^ cycle where the respective currents in either direction near overlap. Also, current increases progressively with CV cycle or time as shown is Figure S6 (Supporting Information). This can be explained by stress amplification induced by either or both dislocation climb creep (DCC) and glide assisted plastic flow of lithium at the depleted contact, effected during stripping. This is discussed in detail in the next section. Figure 3d shows the magnified view of the interface during low (<5 mV) and high (>5 mV) anodic overpotentials corresponding to the linear and non‐linear *I–η* regions, respectively. Clearly, Figure 3d indicates the decrease in contact area with potential. Clearly, increasing anodic overpotentials lead to progressively depleting contacts with concurrently increasing contact stresses via stress amplification.

To gain further insight into the influence of interfacial contact on *I–η* behavior, an asymmetric potential sweep in the CV was performed. Figure 3b shows an asymmetric CV with anodic sweep potential (+20 mV vs Li^+^/Li) twice that of the cathodic sweep (−10 mV vs Li^+^/Li), i.e., more charge is stripped than plated. Such an experiment was conducted so as to cause insufficient time and contact for interface recovery (discussed next). As before, overlap between *I*_cat_(forward) and *I*_cat_(reverse) is observed during the first cathodic scan. When switching to anodic scan, current hysteresis is induced, i.e., *I*_an_(forward)> *I*_an_(reverse)_._ This effect continues in the second cycle of the cathodic scan where it can be seen that *I*_cat_(forward)< *I*_cat_(reverse). Thereafter, a decrease in current (compared to first cycle) along with the overlap between the forward and reverse current is observed. Figure S7 shows the asymmetric CV at 0.5 mV s^−1^. A behavior as in Figure 3b is observed, with a pronounced current hysteresis starting at the 4^th^ cycle.

### 2.4 Creep in the Microelectrode

The lattice rigidity of ISE leads to a different LiǀISE interfacial structure compared to LEs. The diffusion limitation in Li leads to a loss of interfacial contact during stripping as depicted in Figures 3c,d and is responsible for the current hysteresis (Figure 3a).^[^ ^11^, ^48^ ^]^ The subsequent closure of the hysteresis in Figure 3a is due to interfacial contact healing and can be attributed to the creep and glide assisted plastic flow at the depleted contacts via high contact stresses.^[^ ^49^ ^]^

Creep is a time‐dependent plastic deformation occurring at high homologous temperatures (i.e., *T*_H_ > 0.3‐0.4, and *T*_H_ (= *T*/*T*_fus_) for Li is 0.65, where *T* and *T*_fus_ (= 453.65 K) are the temperature of the experiment and melting temperature of Li in Kelvin, respectively) and below yield stress. In addition, creep depends on the microstructure, i.e., grain size, dislocation density, etc. The general expression for steady‐state creep is given by^[^ ^33^ ^]^

(4) $$\dot{\varepsilon }=\frac{AD_{i}Gb}{kT}\;{\left(\frac{b}{d}\right)}^{r}{\left(\frac{\sigma }{G}\right)}^{p}$$

where $\dot{\varepsilon }$ is the strain rate, *A* is a material‐specific constant, *D_i_* is the self‐diffusivity (“*i*” can either denote bulk/grain boundary diffusivity or pipe diffusion), *G* is the shear modulus, *b* is the length of the Burgers vector, *k* is Boltzmann constant, *T* is the absolute temperature, *d* is the grain diameter, *σ* is the applied stress, and *r* and *p* denote grain size exponent and stress exponent, respectively. Creep in metals occurs primarily either via diffusional creep and/or dislocation or power law creep.^[^ ^49^, ^50^ ^]^ Diffusional creep occurs at low stresses, exhibits a linear relationship between strain rate and stress, and is strongly dependent on grain size. For fine‐grained polycrystalline samples, grain boundary diffusion dominates (Coble creep), while for coarse‐grained samples, volume diffusion is the main pathway (Nabarro–Herring).^[^ ^49^ ^]^ Whereas dislocation creep occurs at higher stresses and is characterized by a power law dependence on stress.

### 2.5 Deformation at Low Overpotential (<5 mV)

**Figure 4:**
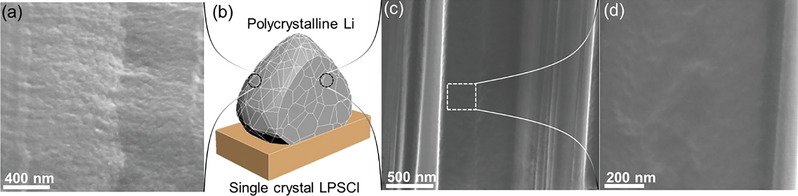
a) SEM image showing the surface topography of a Li microelectrode of a region indicated in the schematic (b). c) SEM image of a FIB cut region, as shown in (b). d) High magnification image of dotted box region in (c).

This corresponds to the linear *I‐η* regime in Figure 3a pertaining to a small fraction of extracted charge and hence a relatively large fraction of contact area. To understand the possible influence of lithium microstructure on creep, high magnification SEM image of the microelectrode was acquired (**Figure** **4**a). Surface topography suggests a fine‐grained (<100 nm) polycrystalline microstructure of the electrodeposited lithium, as shown in Figure 4b. To correlate surface topography with the inner microstructure, SEM images of a focused ion beam (FIB) milled region, as indicated in Figure 4b were acquired (Figure 4c,d). Figure 4d is the magnified view of the dotted area in Figure 4c. Although it is difficult to determine the exact grain size, the contrast in Figure 4d may possibly be due to different grain orientations.

Thus, fine‐grained polycrystalline microstructure suggests that the Coble creep, assisted by low activation grain boundary diffusion (indicated by solid circles in Figure 3d), might possibly be the predominant deformation mechanism, as $\dot{\varepsilon }$ varies with 1/*d* ^3^ according to Equation 5 ^[^ ^48^ ^]^

(5) $$\dot{\varepsilon }=\;\psi \frac{\sigma \Omega_{i}\delta_{\mathrm{GB}}D_{\mathrm{GB}}}{kTd^{3}}$$

*Ω*_i_ is the atomic volume, *δ*_GB_ is the grain boundary width, *D*_GB_ is the diffusivity along the grain boundary, and *ψ* = 14*π* is a constant. The deformation in a representative volume element is indicated by the dotted circle in Figure 3d is shown in Figure 3e. The contour of an initial undeformed grain (indicated in red) is superimposed over the deformed grain.

### 2.6 Deformation at High Overpotential (>5 mV)

**Figure 5:**
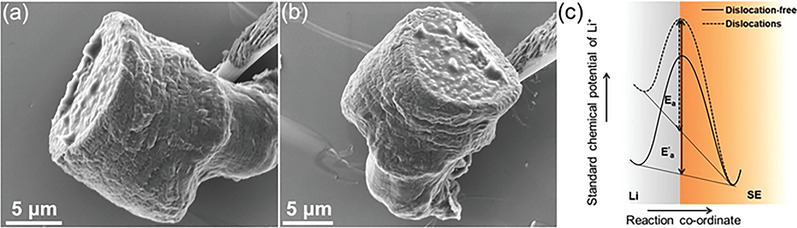
SEM images of stripped microelectrode surfaces from different perspectives, a) side and b) top view. b) Schematic indicating interfacial charge‐transfer barriers for the elementary reaction Li → Li^+^ + e^−^ in the presence and absence of dislocations in the lithium anode. The energy barriers are indicated over the weighted mean of the standard chemical potential of the two end states.

This corresponds to the non‐linear *I–η* regime in Figure 3a pertaining to a large fraction of extracted charge and hence a relatively small fraction of contact area. **Figure** **5**a,b shows SEM images of an inverted microelectrode, stripped at ≈41 mA cm^−2^ (discussed later in detail). An inhomogeneous stripping is observed. Edges are slightly protruded whereas the other regions indicate nearly uniform stripping. This implies a load inhomogeneity induced stress amplification at the edges due to finite needle stress.^[^ ^51^, ^52^ ^]^ Therefore, power law creep or dislocation climb creep (DCC) or glide assisted plastic flow is expected to be operative at higher anodic overpotentials leading to progressively increasing strain rates (Figure 3e). We refer to a recent work wherein LiǀLLZO interface exhibited a voltage fluctuation during stripping under moderate applied stress (0.2 MPa). It was attributed to stress amplification at the depleted contact leading to glide assisted plastic flow in Li.

Therefore, evaluating the nature of the lithium microstructure is crucial for the development of anode‐free cells wherein the microstructure governs the interface instability. Also, it controls the subsequent morphology during discharge and is discussed next.

### 2.7 Stripped Microelectrode Morphology and Effect of Microstructure on Kinetics

SEM images of the stripped microelectrode interface (see Figure 5a) indicates nearly uniform stripping except at the edges which appear to be slightly protruded. This is contrary to the observed stripped LMA morphology in literature wherein micron‐sized pores are observed.^[^ ^11^ ^]^ A mechanism is proposed below to explain the observed morphology.

**Figure 6:**
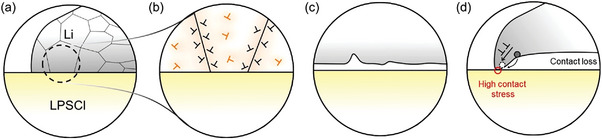
Schematic indicating the effect of microstructure on the morphology of the stripped microelectrode. a) Fine‐grained polycrystalline microelectrode. The dotted area in (a) is expanded in (b), indicating the uniform distribution of free and GB dislocations. Orange, free dislocation; black, GB dislocation. The dislocation strain field is shaded in light orange. c) Uniform distribution and high dislocation density lead to uniform Li stripping at the interface. d) High contact stresses at the periphery lead to a change in the deformation mechanism from DCC to slip‐assisted flow. This allows a higher fraction of charge transfer through this region. Mass transport by surface diffusion (represented by the curved arrow) fills the vacancy (dotted circle) formed at the interface.

Figure 4 indicates that our microelectrodes have a fine‐grained polycrystalline microstructure indicating a high density of GBs. Therefore, the microstructure can be approximated as showing a high dislocation density, as grain boundaries can in principle be considered as an array of dislocations to a first approximation (**Figure** **6**a,b).^[^ ^53^ ^]^ The microelectrode can additionally be expected to be strain hardened^[^ ^54^ ^]^ under needle stress during its growth (the needle is displaced during its growth, Figure 1d). Thus, indicating a significant dislocation population in the grain interior (6b). Therefore, the microstructure can be hypothesized to have a uniform and dense distribution of dislocations.

Shishvan et al. have proposed that interfacial kinetics gets modified in the presence of dislocations as shown in Figure 5c. It is proposed that the dislocations in the lithium, via lattice distortion, influence the interfacial kinetics in two basic ways. Firstly, in the vicinity of the dislocations, the lattice expansion leads to an increase in effective vacant sites (${\hat{\theta }}_{V}$) and is given by^[^ ^55^ ^]^

(6) $${\hat{\theta }}_{V}=e^{-\frac{H_{V}}{RT}}\;+\alpha \frac{\Omega_{\mathrm{Li}}\left(\rho_{d}b^{2}\right)}{\Omega_{V}}$$

where *H*_V_ is the enthalpy of vacancy formation, *Ω*_Li_ and *Ω*_V_ are the molar volumes of lithium and vacancies respectively, *ρ*_d_ is dislocation density, *α* is a constant and depends on the crystal structure of the metals, *b* is the magnitude of the Burgers vector, *R* and *T* are universal gas constant and absolute temperature, respectively. The second term on the right‐hand side of the above equation accounts for the additional space created due to the lattice expansion around the dislocations. Secondly, lattice disorder and distortion, in the core and around the dislocation, enhance the enthalpy of Li^+^ ions in the electrode.

The increase in standard chemical potential of Li^+^ ions in the electrode leads to a reduction of the charge transfer barrier across the interface (Figure 5c) and is shown to be^[^ ^55^ ^]^

(7) $$R_{\mathrm{CT}}=R_{0,\mathrm{CT}}\;{\hat{\theta }}_{V}^{\beta -1}e^{-\frac{\left(1-\beta \right)H_{V}}{RT}}$$

where *R*_CT_ and *R*_0,CT_ are the charge‐transfer resistances in the presence and absence of dislocations and applied stress respectively, ${\hat{\theta }}_{V}$ is the effective vacant sites and *β* is the symmetry factor. It has been shown that for *β* = 0.5, *R*_CT_ < *R*_0,CT_ for increasing dislocation density.

We note that interfacial charge‐transfer reactions, apart from energetics, must also consider mass‐transport in the adjacent phases. Clearly, regions in and around dislocations are associated with higher diffusivity than in the bulk (dislocation pipe diffusion or grain boundary diffusion).^[^ ^56^ ^]^ Therefore, we conclude that these microelectrodes, on account of the uniform and dense distribution of dislocations, show nearly uniform stripping (Figure 6c). Figure 6d shows the situation at the edges at high overpotential. The protruded morphology might possibly be related to enhanced mass transport along the external electrode surface, influenced by surface tension effects at the three‐phase boundary and interface.

### 2.8 Mechanical Endurance of Grain Surfaces Versus Grain Surface Flaws Towards Dendrite Growth

To gain insight into the preferential dendrite growth pathways in the LPSCl microstructure, lithium was selectively deposited at i) a flaw‐free grain surface (GS) and ii) a preexisting grain surface flaw (GF) (crack‐like defect). This is particularly important for LPSCl since the formation of interfacial contacts is associated with a significant “flaw density” due to the low *K*_IC_ for sulfide‐based ISEs.^[^ ^19^ ^]^

**Figure 7:**
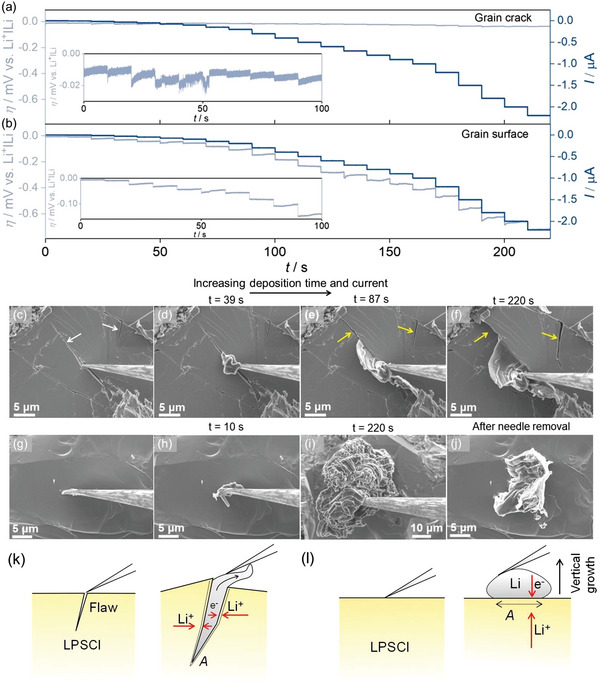
Chronopotentiometric profiles for lithium deposition on a a) flawed grain surface and b) flaw‐free grain surface region. Insets in (a) and (b) show magnified data of the initial overpotentials. c) SEM image showing the tungsten needle contacted at a flawed surface region prior to lithium deposition whereas d) shows the lithium deposition at t = 39 s. e,f) SEM images of the temporal evolution of the lithium insertion driven LPSCl fracture. Arrows indicate the crack extension. g) SEM image of a flaw‐free grain surface in contact with the tungsten needle prior to the deposition. h,i) SEM images showing preferential vertical growth of lithium with time. j) SEM image of (g) after galvanostatic cycle. k) Schematic indicating lithium insertion driven fracture. l) Vertical growth of lithium occurs on a flaw free grain surface region. The dotted lines in (k) and (l) indicate the contact area. The curved arrow indicates free growth of lithium above the surface of LPSCl.

Prior to measurements, the tungsten microelectrode was pre‐lithiated at −2 nA for 45 s. Then, under identical galvanostatic conditions, the potential profiles shown in **Figure** **7** were recorded. The galvanostatic sequence included an initial current of −2 nA followed by increments up to −2.2 µA with a step duration of 10 s each (Figure 7a,b). Figure 7a,b shows that the evolution of the overvoltage is different for GF and GS contacts. The overvoltage increases only slightly between current steps when deposition occurs at the GF contact, in contrast to the GS contact, where the increase is more pronounced. For example, the insets in Figure 7a,b show that the overpotential ǀ*η*ǀ after 100 s with an applied current of −0.3 µA in the GF case is 15 mV, which is nine times smaller than the GS deposition overpotential of 137 mV. The corresponding ǀ*η*ǀ values at 220 s and an applied current of −2.2 µA were 31 mV and 715 mV for the deposition at GF and GS contacts, respectively.

Figure 7c,g shows the GF and GS regions contacted with the microneedle before deposition. Figure 7d–f shows that the temporal evolution during galvanostatic deposition at the GF contact is accompanied by the fracture of the contacted SE (indicated by yellow arrows). In contrast, deposition at the GS contact (Figure 7h–j) leads to vertical lithium growth without fracture. Similarly, deposition at a grain boundary (GB) leads to fracture as in the case of deposition at the GF contact (Figure S8, Supporting Information). As Young's modulus of grain boundaries in ceramics is usually one to two orders of magnitude lower compared to that of grain interior, the reduced stiffness of the grain boundaries is one possible reason for the preferential lithium growth along these regions,^[^ ^18^, ^57^, ^58^ ^]^ while electronic effects may also add another reason.^[^ ^59^, ^60^ ^]^ This clearly supports that GF and GB are the preferred regions for dendrite propagation in the LPSCl microstructure and are the primary causes of failure in SSBs, which is reflected in the observed low CCD values.

The observed different overpotentials (Figure 7a,b) can be considered to be caused by the differences in constriction impedance, as *Z*_constriction_ ∝ 1/*d*, Equation 1.^[^ ^26^, ^27^, ^28^ ^]^ Lithium growth inside the crack (Figure 7e,f) results in a larger contact area because the opposite surface of the crack is in contact with Li, resulting in a larger *d*, in contrast to deposition on a GS region, where vertical growth results in a small constant contact area and thus a smaller *d*. This can be seen from the comparison between Figure 7i,j. Therefore, *Z*_constriction_ is higher for deposition in GS regions, leading to a higher overpotential (Figure 7b).

Deposition at a GF region leads to the filling of the crack. Theoretically, it has been shown that further deposition can build up compressive stresses of ≈1 GPa for lithium, well above its yield strength (0.8 MPa).^[^ ^12^, ^14^, ^61^ ^]^ Lithium in this state can be considered as having no strength and behaves like an incompressible and inviscid fluid exerting a uniform normal stress on the crack walls.^[^ ^14^ ^]^ Further deposition leads to a stress intensity factor (*K*_I_) that exceeds the fracture toughness (*K*_IC_), resulting in fracture (Figure 5c–f,l).^[^ ^14^ ^]^

(8) $$\sigma_{c}\geq \frac{K_{\mathrm{IC}}}{Y\sqrt{\pi a}}$$

where *K*_IC_ is the fracture toughness of the SE, *Y* is the geometric factor, and *σ*_c_ is the critical stress for a given flaw size *a*. Crack extension causes unloading of the crack tip and the process repeats until the critical stress for the newer crack geometry is rebuilt. As shown in Video S1 (Supporting Information), fracture accelerates with time and thus with applied current, since the rate of pressure build‐up in the crack is proportional to the applied current density and given by^[^ ^14^ ^]^

(9) $$\frac{dp}{dt}=\;-\frac{0.44E^{\prime \prime }\Omega_{M}i}{\mathrm{aF}}$$

where d*p*/d*t* is the pressure build‐up rate, *E*′′ = *E´*/(1−*ν* ^2^); *E´* and *ν* are Young's modulus and Poisson ratio of the SE, *Ω*_M_ is the molar volume of lithium (13.02 cm^3^ mol^−1^), *i* is the current density, *a* is the crack length, and *F* is Faraday's constant.

### 2.9 Rate Capability at the Microelectrode

**Figure 8:**
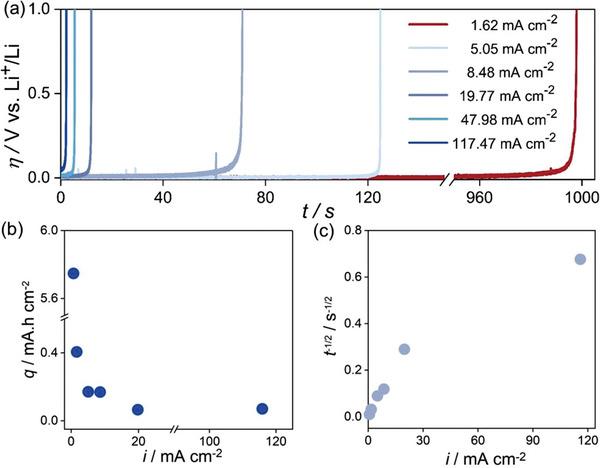
a) Rate capability behavior of the microelectrode. b) Charge extracted versus current density. c) *t* ^−1/2^ versus current density.

Following the mechanical endurance test, we evaluated the rate capability of the microelectrode (**Figure** **8**a). A decrease in time to reach the limiting voltage, i.e., 1.0 V, is observed with increasing current density, as expected. Figure 8b shows the plot of extracted areal charge versus current density. The extracted charge decreases rapidly with increasing current density from ≈1.62 mA cm^−2^ to ≈8.48 mA cm^−2^. Only a slight decrease is observed at ≈19.77 mA cm^−2^. Another decrease is observed at 47.88 mA cm^−2^, and thereafter the extracted charge becomes independent of the current density (Figure 8b).

Thereafter, in order to evaluate the Li self‐diffusion coefficient, a graph of *t* ^−1/2^ versus *i* was plotted (Figure 8c) according to Sand's equation^[^ ^62^ ^]^

(10) $$t^{-1/2}=2i/\left(\sqrt{D\Pi }c_{0}F\right)$$

where *t* is the transition or depletion time (or the time required to reach cut‐off voltage of 1.0 V, Figure 8a), *i* is the current density, *D* is the self‐diffusion coefficient of lithium, *c*_0_ is the concentration of lithium (76.8 mmol cm^−3^) and F is the Faradays constant. Equation 10 predicts a linear relationship between *t* ^−1/2^ and *i*, contrary to the observed behavior (Figure 8c). Figure 8c shows an ever‐increasing diffusivity with increasing current density. Clearly, this cannot be true unless the temperature rises significantly during the measurements. The plausible reason for the observed apparent discrepancy is discussed next.

The theoretical basis of Equation 8 is the linear 1D diffusion profile derived by Fick's second law.^[^ ^62^ ^]^ However, in general, diffusion in solids is anisotropic which is reflected by the symmetric second rank diffusion tensor. Although the diffusion tensor is isotropic for cubic systems, (i.e., bcc for Li), complications arise depending on the microstructural state of the solid, i.e., grain size, dislocation density, etc., since self‐diffusion follows the following order *D*_S_ ≥ *D*_GB_ ≥ *D*_D_ > *D*_V_, where the subscripts denote: S, grain surface; GB, grain boundary; D, dislocations; and V, bulk.^[^ ^56^ ^]^ Therefore, their spatial distribution and density do neither guarantee a flux vector normal to surfaces of the same concentration, nor do they guarantee spatially uniform diffusivity. This is particularly relevant in the currently prevailing GB microstructure. This leads to the breakdown of the underlying assumptions used to derive Equation 10. In fact, diffusivity measurements are ideally performed on well‐annealed single crystals having low non‐equilibrium defect density. Therefore, a more rigorous treatment is required to account for the directional relationship between the concentration gradient and the flux vectors.

In liquid electrolytes, where ions are dispersed in a homogenous dielectric, consumption of the species sets up a concentration gradient with time. A similar concentration gradient cannot be set up inside a metal; this is equivalent to dilution or formation of the free surface. Instead, defects created due to electrodissolution are restricted to the top atomic layer only. This can be one of the reasons for the observed disparity in solid‐state systems.

In addition, spatially varying dislocation‐induced kinetics and associated diffusivities lead to inhomogeneous stripping and thus to a spatially varying interfacial contact (Figure 5). This means that the flux increases progressively with time. This is again a violation of the assumptions underlying Equation 10.

## 3 Conclusion

We investigated the polarization behavior of the Li|LPSCl interface at high current densities using a lithium microelectrode setup. Typically, this regime, which is relevant for the development of fast‐charging SSBs, is inaccessible in pouch cell measurements due to low CCD values. Using the microelectrode approach, we demonstrate that the Li|LPSCl interface exhibits non‐linear kinetics. In line with studies of Liǀpolymer interface, we attribute this non‐linearity to the strong electric field across the thin, disordered SEI at high current density. Based on previous reports of non‐linear SEI kinetics, we hypothesize that there are several rate‐limiting processes, i.e., charge transfer across Li|SEI and SE|SEI interfaces together with ion transport across the SEI, which together determines the non‐linear interface kinetics observed at high current density.

We also highlight the importance of the lithium microstructure on creep and kinetics. We show that nanocrystalline lithium can lead to a stable LiǀSE interface by grain boundary assisted creep even at high current density. This indicates that fine‐grained polycrystalline lithium can reduce the required pressure in SSBs. In addition, a dense grain boundary microstructure leads to uniform stripping via a high density of non‐equilibrium defects having a reduced charge‐transfer barrier. This would lead to a further reduction in stack pressure by alleviating the problems of interfacial loading inhomogeneity. However, fast grain growth at ambient temperatures will lead to strong transient behavior of the lithium anode after plating.

Due to the low fracture toughness of sulfide‐type SEs, interfacial contact is mediated by their fracture. Therefore, Li|LPSCl interfaces exhibit a significant defect population whose role in dendrite growth is important. We show by local deposition of lithium on defect‐free grain surfaces, grain defects, and GBs that it is the latter two that facilitate dendrite growth in LPSCl. This is important for the development of “anode‐free” SSBs because the stability of the interface is determined by the defect population, stress generated at the interface, and on the microstructure of the deposited lithium. Finally, we discuss the pitfalls of the conventional Sand's equation in deriving self‐diffusion coefficients in solid‐state systems.

## Conflict of Interest

The authors declare no conflict of interest.

## Author Contributions

D.K.S. and J.J. conceived the idea. D.K.S. designed the research plan, performed the experiments, and wrote the initial draft. J.J. guided and supervised. D.K.S., C.K., and J.J. analyzed the data. B.M. provided technical help for the in‐situ setup. D.K.S., T.F., C.K., and J.J. were involved in writing, reviewing, and editing. J.J. acquired the funding. All the authors discussed the results and commented on the manuscript.

## Supporting information

Supporting Information

Supplemental Video 1

## Data Availability

The data that support the findings of this study are available from the corresponding author upon reasonable request.
