# Supplementary material for: Non‐Linear Kinetics of The Lithium Metal Anode on Li6PS5Cl at High Current Density: Dendrite Growth and the Role of Lithium Microstructure on Creep
Source: Adv Sci (Weinh). 2023 May 23;10(22):2302521. doi: 10.1002/advs.202302521 (PMC10401129; doi:10.1002/advs.202302521)
Supplement: Supplementary file 1 — Supporting Information [file ADVS-10-2302521-s002.pdf]

ADVANCED SCIENCE
Open Access

Supporting Information

for *Adv. Sci.*, DOI 10.1002/advs.202302521

Non-Linear Kinetics of The Lithium Metal Anode on Li_6PS_5Cl at High Current Density: Dendrite Growth and the Role of Lithium Microstructure on Creep

Dheeraj Kumar Singh, Till Fuchs, Christian Krempaszky, Boris Mogwitz and Jürgen Janek**

Supplemental Notes

Non-linear kinetics of the Lithium Metal Anode on Li_6PS_5Cl at High Current Density − Dendrite Growth and the Role of Lithium Microstructure on Creep

Dheeraj Kumar Singh,[1,2] Till Fuchs, [1,2] Christian Krempaszky,[3] Boris Mogwitz,[1,2] and Jürgen Janek[1,2 *]

[1]Institute of Physical Chemistry, Justus-Liebig-University Giessen, Heinrich-Buff-Ring 17, D-35392 Giessen, Germany.

[2]Center for Materials Research (ZfM), Justus-Liebig-University Giessen, Heinrich-Buff-Ring 16, D-35392 Giessen, Germany.

[3]Institute of Materials Science and Mechanics of Materials, Technical University of Munich, Boltzmannstrasse 15, D-85748 Garching, Germany.

*Corresponding author: dheeraj.interfaces@gmail.com, juergen.janek@pc.jlug.de

Experimental section

Synthesis of Li_6PS_5Cl (LPSCl) LPSCl was prepared using solid-state approach. Prior to synthesis, LiCl, was dried under dynamic vacuum at 723 K for 48 h to remove the majority of H_2O from LiCl·(H_2O). Thereafter, the starting precursors viz., Li_2S, P_2S_5, and LiCl, were mixed in the stoichiometric ratio and then hand-ground in an agate mortar for at least 30 min. Subsequently, the obtained mixture was pressed into a pellet and then transferred into a quartz ampule (12 mm inner diameter and 8–10 cm in length). The quartz ampule was carbon-coated using acetonitrile and then preheated at 1073 K for 2 h under dynamic vacuum to remove all trace of water and functionalized surface moieties in the reaction atmosphere. The quartz ampule was sealed under the vacuum (~ 10^{-3} mbar). Sealed quartz ampule was annealed at 823 K for 1 week to complete the reaction. The final sample was hand-ground into a powder for further analyses.

Preparation of LPSCl pellet Briefly ~240 mg of LPSCl powder was cold compacted into cylindrical pellet using a 10 mm die. Thereafter, it was vacuum sealed using rubber balloon (CPR GmbH, Sonodomen). It was further reinforced with additional two more layers of rubber, thereafter, the pellet was isostatically compressed at 362 MPa for 30 min. The obtained pellet had a density ~92%.

Experimental set-up for in-operando SEM studies: The set-up implemented to study in-operando Li microelectrode behaviour is schematically shown in Figure 1a. Briefly, Li (⌀ = 6.00 mm) was isostatically appended at 265 MPa to one side of the LPSCl pellet which served as a both the reference and the counter electrode. Thereafter, the obtained Li appended pellet was cleaved into nearly two equal semi-circular halves in a direction perpendicular to Li surface to obtain predominantly transgranularly fractured LPSCl surface as shown in Figure 1c and Figure S1. Subsequently, half cleaved pellet was loaded onto an aluminium holder and positioned via set screw (Figure 1a and Figure S4). The in-operando set-up was fitted with a micromanipulator tungsten needle having xyz control for lithium deposition.

The lithium deposition was performed at the tungsten needle on a transgranularly fractured, LPSCl grain surface. A typical deposition profile involved following sequence: -2 nA for 10 min and -3 nA for 50 mins followed by -4 nA 30 mins (Figure 1 c). The deposition was performed at very small currents to avoid current focussing induced cathodic fracture. Additionally, increasing deposition current resulted in the irregular and variable deposition morphology. We observed that implementing such a current sequence resulted in consistent morphology i.e., prolate hemispheroidal deposits with nearly similar contact areas (Figure 1d). This additionally ensured that Li is in the similar stress state across the deposits. Figure 1 d shows the potential profile for the electrodeposition under above mentioned current sequence. A large initial increase in cathodic potential (~-800 mV) is attributed to the nucleation barrier of lithium on tungsten (Figure 1c). Thereafter, Li deposition occurs at a much smaller (~-5 mV) potential.

Material characterization: The phase identification of LPSCl was carried out using powder X-ray diffraction (PXRD) analysis. X-ray diffraction measurements were carried out on the PANalytical Empyrean powder diffractometer in Bragg−Brentano θ-θ geometry with Cu Kα radiation. Measurements were carried out in the 2θ range between 10° and 90° with a step size of 0.026°. The counting time per step was 300 s. All powders were placed on (911) oriented silicon zero background.

Electrochemical measurements: 200 mg of SE was initially cold compacted into cylindrical pellets using hand applied stress through a 10 mm die. Subsequently, the obtained pellet was vacuum sealed in a rubber balloon (CPR GmbH, Sonodomen). Additional two more protective rubber layers were added to ensure adequate precaution for vacuum reinforcement and rupture damage. All the experiments were carried out in Ar filled glove box ($p(O_2)/p$ < 0.5 ppm and $p(H_2O)/p$ < 0.5 ppm). Thereafter, the pellets were isostatically compressed at 362 MPa for 1 h. The density of the obtained pellet was found to be ~92%. Thereafter, Li foils of ~115-120 µm thickness were obtained from Li rods (Rockwood Lithium GmbH) post mechanical work-up and cleaning-up process. Symmetrical Li|Li_6PS_5Cl|Cl cells were then fabricated by appending 6 mm circular disks of Li on either face of SE. To reduce interfacial contact resistance, the symmetrical systems were further subjected to three layers of vacuum reinforced sealing process and was isostatically pressed at different pressures viz. 20, 120, and 265 MPa. Thereafter, pouch cells were fabricated and was used for impedance analyses (GEIS) using VMP300 (Biologic Science Instruments) in the frequency range of 2 MHz to 1 Hz with 10% amplitude of the applied current.

Ionic conductivities were measured by AC impedance spectroscopy using pellets with a thickness of ~2.2 mm and geometric density ~83 % with vapor-deposited gold layers. The as-synthesized powder was pelletized by isostatic pressing at 365 MPa. Then gold layers deposited ~ 200 nm thickness were deposited on both sides. The obtained pellets were sealed under the Ar atmosphere in pouch cells using aluminum as the current collector for the transport measurements. Electrochemical impedance spectroscopy (EIS) was conducted in the temperature range of 233 K to 333 K (Weiss Klimatechnik Climate Chamber) using a VMP300 impedance analyzer (Biologic Science Instruments) at frequencies from 7 MHz to 100 mHz with an amplitude of 10 mV.

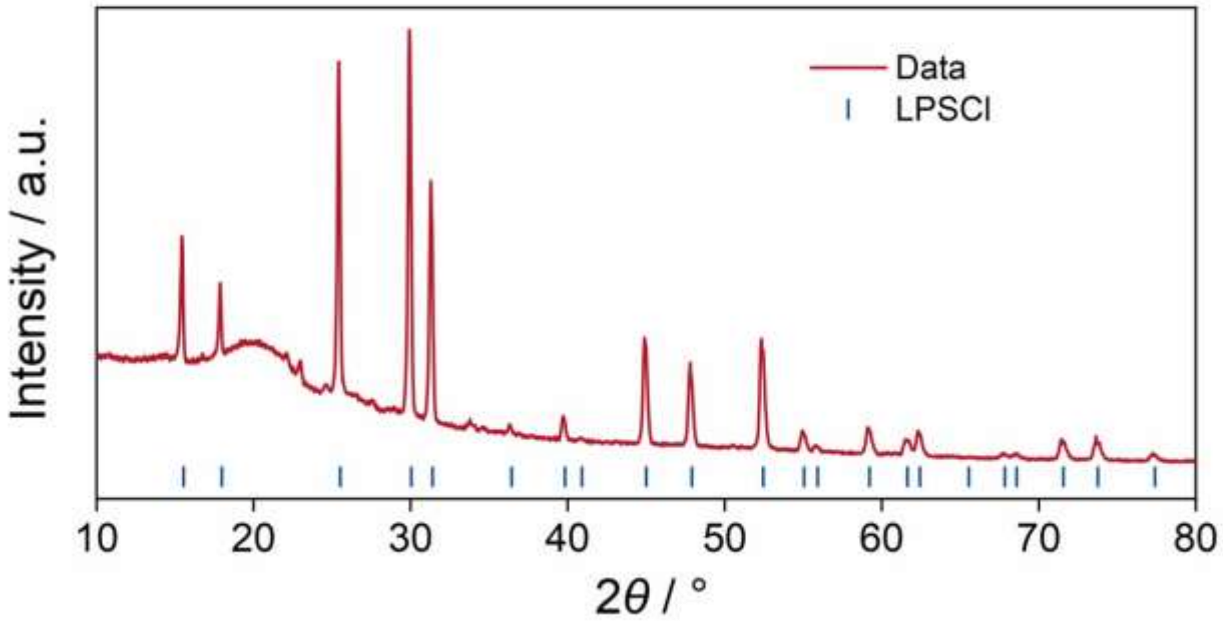

Figure S1. Powder X-ray diffraction (PXRD) pattern of LPSCl.

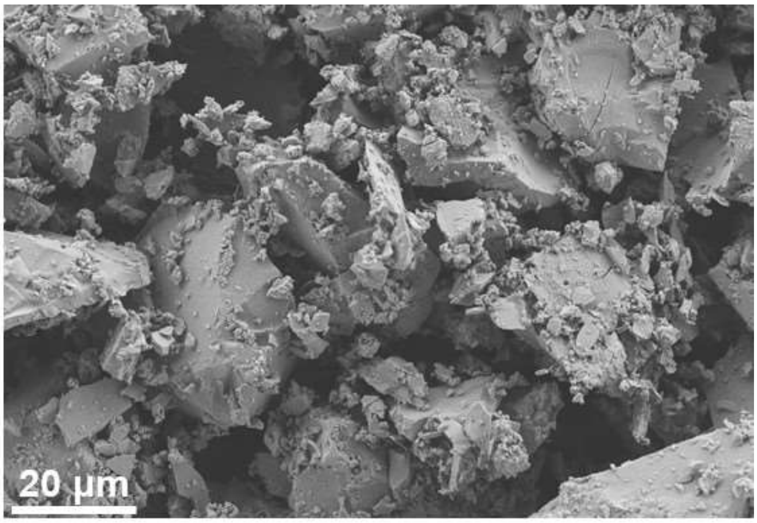

Figure S2. Scanning electron microscopy (SEM) image of LPSCl powder.

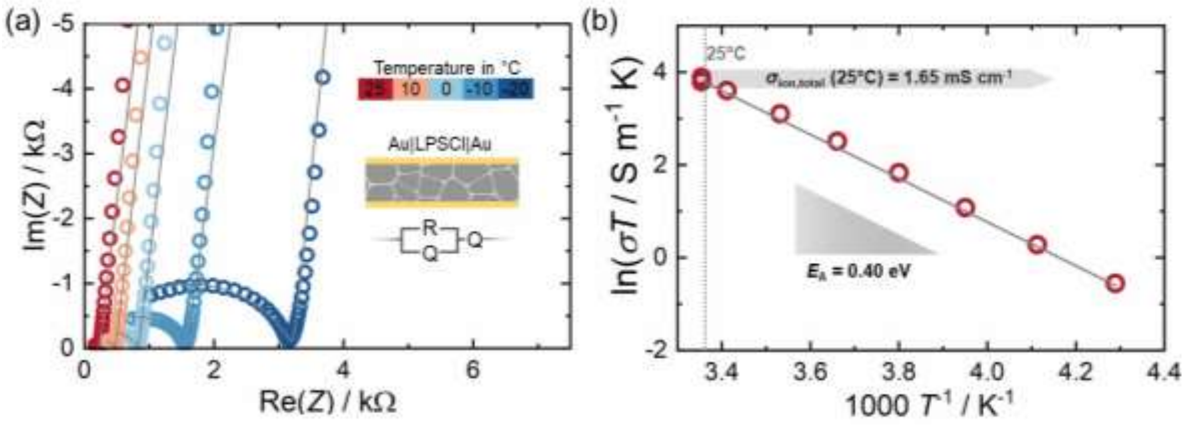

Figure S3. Ion-transport measurements. (a) Nyquist plots at different temperatures (open circles), measured in a symmetric Au|LPSCl|Au cell, fitted using the equivalent circuit shown in the inset. (b) Arrhenius plot for the determination of activation energy (E_A).

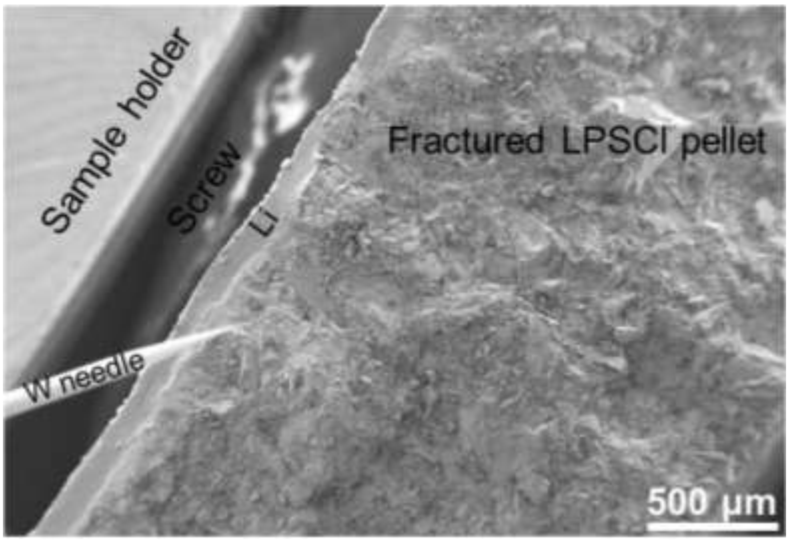

Figure S4. SEM image (top view) of the set-up used for microelectrode investigation.

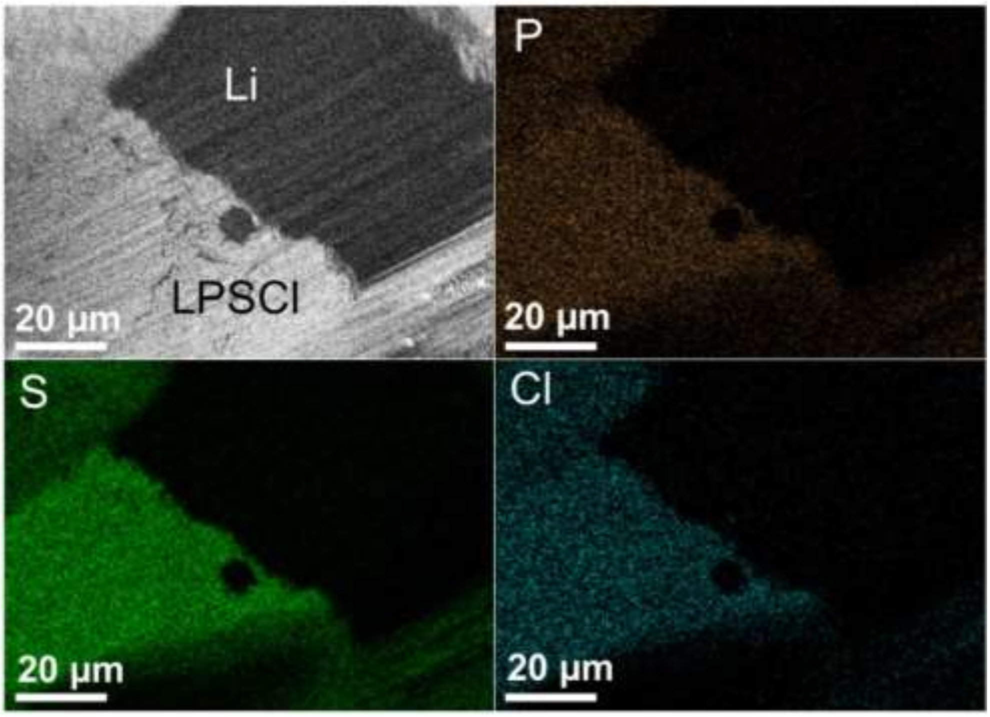

Figure S5. FIB-SEM image of the Li|LPSCl interface and the corresponding energy dispersive X-ray spectroscopy (EDS) elemental mapping of P, S, and Cl.

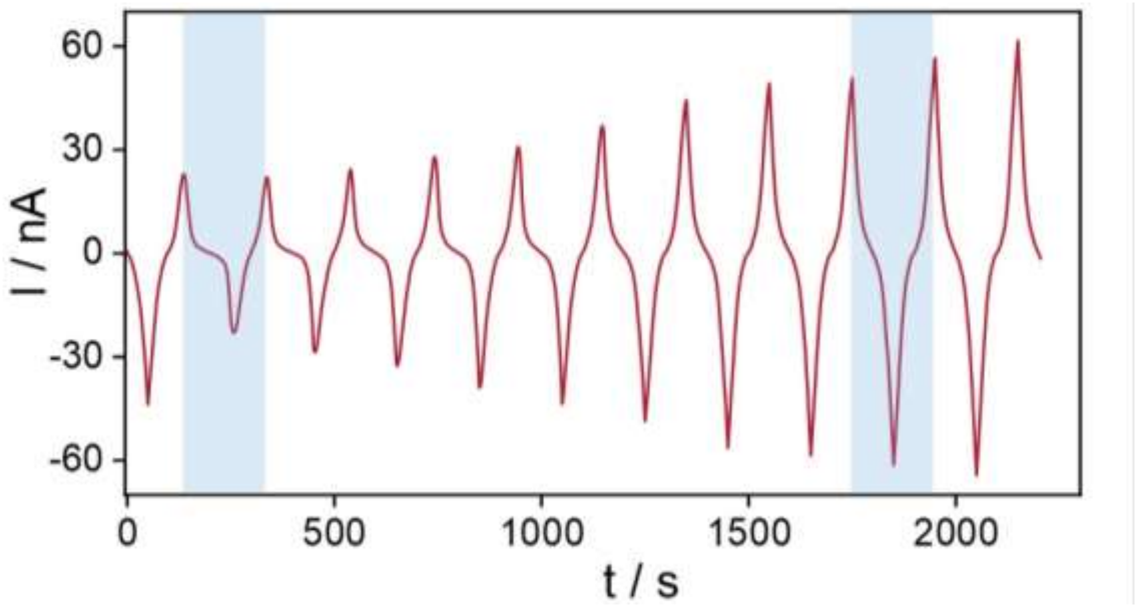

Figure S6. Current vs. time plot of the CV performed at 0.2 mV s^{-1} (Figure 3a).

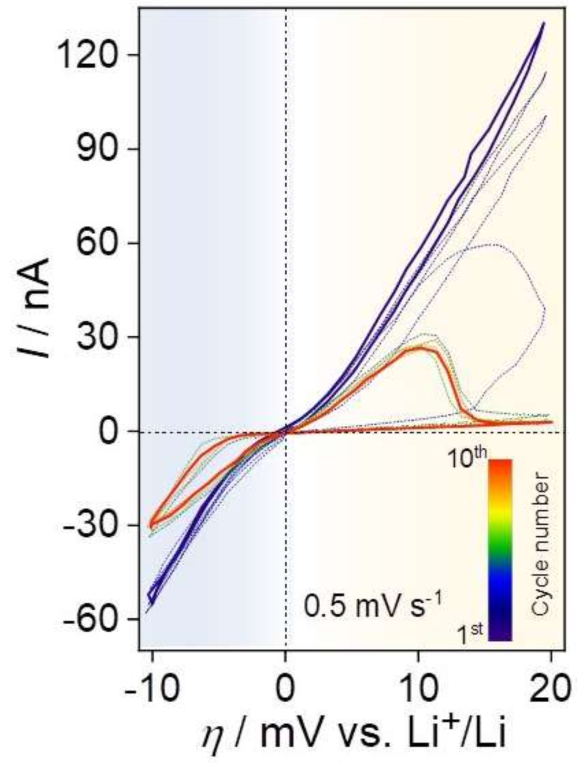

Figure S7. Asymmetric CV at 0.5 mV s^{-1}.

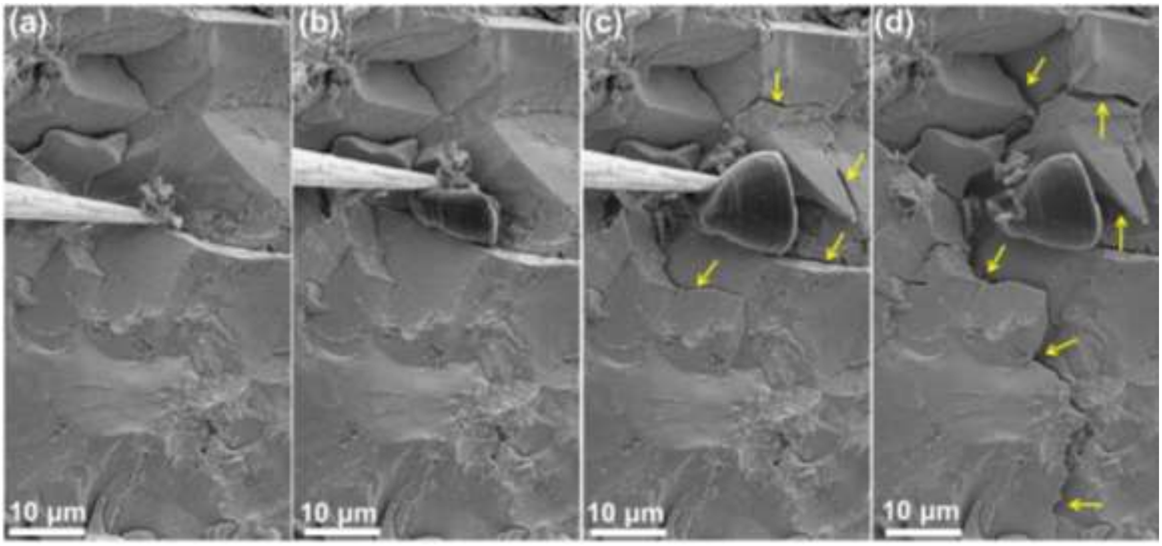

Figure S8. Selective deposition at the grain boundary (GB). (a) SEM image indicating tungsten needle contacted at the grain boundary prior to deposition. (b-c) Temporal evolution of the deposition. Lithium insertion along the GB leads to fracture of LPSCl as indicated by the yellow arrows in (c). (d) Crack extension at the end of deposition.
